# CRISPR-Cas-Integrated LAMP

**DOI:** 10.3390/bios12111035

**Published:** 2022-11-17

**Authors:** Nazente Atçeken, Defne Yigci, Berin Ozdalgic, Savas Tasoglu

**Affiliations:** 1 Koç University Translational Medicine Research Center (KUTTAM), Koç University, Istanbul 34450, Turkey; 2 School of Medicine, Koç University, Istanbul 34450, Turkey; 3 Department of Mechanical Engineering, Engineering Faculty, Koç University, Istanbul 34450, Turkey; 4 School of Medical Services & Techniques, Dogus University, Istanbul 34775, Turkey; 5 Boğaziçi Institute of Biomedical Engineering, Boğaziçi University, Istanbul 34684, Turkey; 6 Koç University Arçelik Research Center for Creative Industries (KUAR), Koç University, Istanbul 34450, Turkey

**Keywords:** loop-mediated isothermal amplification (LAMP), clustered regularly interspaced short palindromic repeat (CRISPR)-associated (CRISPR-Cas), CRISPR/Cas combined LAMP technology, point-of-care (PoC) platform

## Abstract

Pathogen-specific point-of-care (PoC) diagnostic tests have become an important need in the fight against infectious diseases and epidemics in recent years. PoC diagnostic tests are designed with the following parameters in mind: rapidity, accuracy, sensitivity, specificity, and ease of use. Molecular techniques are the gold standard for pathogen detection due to their accuracy and specificity. There are various limitations in adapting molecular diagnostic methods to PoC diagnostic tests. Efforts to overcome limitations are focused on the development of integrated molecular diagnostics by utilizing the latest technologies available to create the most successful PoC diagnostic platforms. With this point of view, a new generation technology was developed by combining loop-mediated isothermal amplification (LAMP) technology with clustered regularly interspaced short palindromic repeat (CRISPR)-associated (CRISPR-Cas) technology. This integrated approach benefits from the properties of LAMP technology, namely its high efficiency, short turnaround time, and the lack of need for a complex device. It also makes use of the programmable function of CRISPR-Cas technology and the collateral cleavage activity of certain Cas proteins that allow for convenient reporter detection. Thus, this combined technology enables the development of PoC diagnostic tests with high sensitivity, specificity, and ease of use without the need for complicated devices. In this review, we discuss the advantages and limitations of the CRISPR/Cas combined LAMP technology. We review current limitations to convert CRISPR combined LAMP into pathogen-specific PoC platforms. Furthermore, we point out the need to design more useful PoC platforms using microfabrication technologies by developing strategies that overcome the limitations of this new technology, reduce its complexity, and reduce the risk of contamination.

## 1. Introduction

In infectious diseases and epidemics, the first step in successful disease management is the rapid and accurate detection of the causative agent. The COVID-19 pandemic is an important example of what a pathogen can cause and how it can evolve into a major threat to global public health [1]. It also highlighted the need for point-of-care (PoC) diagnostic tests. The World Health Organization (WHO) has recommended PoC diagnostic tests to be affordable, sensitive, specific, user-friendly, fast and robust, equipment-free, and deliverable to end users (ASSURED) [2]. It is very difficult to create pathogen-specific PoC diagnostic platforms that meet all of these criteria. For this reason, developing combined technologies by taking advantage of existing technologies has been the focus of recent efforts.

Each of the available PoC diagnostic methods has a limitation in at least one criterion. Nucleic acid amplification tests (NAATs) are preferred in terms of accuracy compared to other diagnostic tests, such as serological and immunological assays [3]. Polymerase chain reaction (PCR) is known as the gold standard for accuracy as a molecular diagnostic test. However, it cannot go out of the laboratory environment due to the time-consuming and complicated thermal cycler device requirement [4]. Among NAAT-based tests, isothermal methods provide convenience in their application to PoC diagnostic platforms as these methods do not require complicated devices and equipment. Loop-mediated isothermal amplification (LAMP), one of the NAAT-based isothermal methods, is mostly preferred for pathogen detection [5]. Because it provides high efficiency in a short time and is a modifiable technology, it is the most commonly used isothermal NAAT in PoC diagnostic platforms. LAMP technology was combined with clustered regularly interspaced short palindromic repeats (CRISPR)-associated (CRISPR-Cas) technology to increase the sensitivity and specificity. CRISPR-Cas technology has the potential to specifically detect the targeted nucleic acid sequence because it is programmable and has enzymatic activity. CRISPR/Cas integrated LAMP technology, which can meet the ‘ASSURED’ criteria, allows for the development of new generation PoC diagnostic platforms.

In this review, the modifiability of LAMP technology and the use of CRISPR/Cas technology in nucleic acid amplification detection are described. The advantages and limitations of the two technologies in pathogen detection are examined. CRISPR/Cas combined LAMP technology is explained, and studies on the detection of bacterial and viral pathogens are mentioned. The challenge of converting CRISPR/Cas combined LAMP technology to PoC platforms and the development efforts of PoC devices are discussed.

## 2. LAMP Technology and Its Modifiability

The LAMP method was first developed in 2000 [6] and has been used since then as an excellent tool for pathogen detection. Its popularity has increased with diagnostic use in severe acute respiratory syndrome (SARS) coronavirus [7] and West Nile virus [8]. With the COVID-19 pandemic, many modified LAMP methods have been designed to detect the SARS-CoV-2 virus and LAMP technology has experienced its golden age [9]. By utilizing LAMP technology, pathogen-specific diagnostic tests have been developed for the detection of pathogens that cause many viral, bacterial, fungal, and parasitic diseases [10].

**Figure 1:**
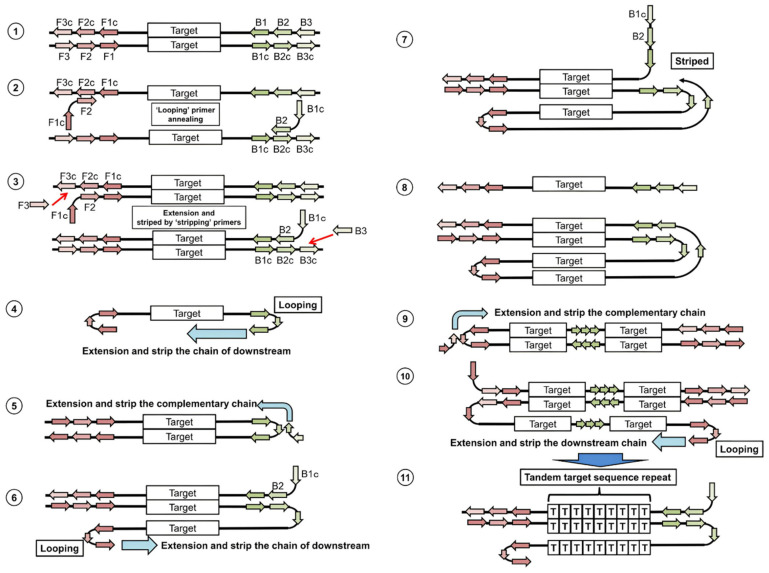
Representation of the LAMP amplification principle in the schematic diagram. Reprinted from Ref. [14].

The LAMP method is one of the isothermal amplification technologies and is a nucleic acid amplification technique (NAAT) [11]. The LAMP principle has a unique primer design using four or six primers [6]. F3, B3 outer primers and FIB (F2, F1 complement), and BIP (B2, B2 complement) inner primers are designed to create a multi-loop amplification product. By designing two loop primers (LF, LB) targeting the loop structures, the amplification product is constituted in the cauliflower structure containing multiple loop structures. As a result, to create amplification with six primers, eight different gene sequences are determined within the targeted gene region, and six different DNA synthesis initiation sites are formed. This primer design logic provides extremely high specificity, and a high rate of amplification product is generated in a short time (Figure 1). The gene region targeted with the LAMP method allows for the easy detection of pathogens found in low concentrations in body fluid samples [12]. For the amplification reaction, the Bst DNA polymerase enzyme is used specifically for the LAMP method. Bst DNA polymerase enzyme has strand displacement activity on the DNA molecule and works optimally at 60–65 °C [6]. Owing to these functional properties of the enzyme, a temperature change to ensure DNA denaturation is not required in the amplification reaction process. LAMP technology attracts attention as a more advantageous technique compared to NAAT-based techniques as there is no need for thermal cycling and amplification reaction occurs under isothermal conditions [5]. The LAMP reaction takes place easily in a hot water bath or a heating block without the need for the complex instrument as used in PCR. The result of the LAMP amplification reaction can be detected using colorimetric, turbidimetric, gel electrophoresis-based, and fluorescence-based methods using many different types of dyes [13]. In addition, the reaction results can easily be observed with the naked eye calorimetrically and turbidimetrically. Thanks to these different types of result monitoring methods, it is possible to modify the LAMP technology for diagnostic purposes in the laboratory environment, field conditions, and as a bedside test [11].

With the LAMP method being a modifiable technology, many Point-of-Care (PoC) diagnostic platforms have been created that are fast, low-cost, easy to use, and compatible with field conditions. Modified LAMP diagnostic platforms have been extremely useful for the diagnosis of infectious diseases, epidemic diseases, neglected diseases [10], and especially during the COVID-19 pandemic [9]. Although there are many pathogen-specific modified LAMP techniques, modified Multiplex-LAMP diagnostic tools have also been designed for the simultaneous analysis of many pathogens (infecting the same tissue or causing similar clinical symptoms) [10]. To detect viral diseases caused by RNA viruses, the reverse transcriptase enzyme was added, and the Reverse Transcriptase-LAMP (RT-LAMP) method was also developed [15]. In addition to pathogen detection by targeting the gene region, various modified LAMP techniques have been developed for single nucleotide polymorphisms (SNP) genotyping and mutation detection for the diagnosis of a particular disease [16,17]. LAMP technology can be modified by using fluorescence probes [18], molecular beacons [19], and nanoparticles [20]. Finally, LAMP technology can be modified into PoC diagnostic platforms combined with CRISPR/Cas technology.

## 3. CRISPR/Cas Technology in Nucleic Acid Amplification Detection

CRISPR-Cas system is a unique and extremely promising technology that allows geneticists and medical researchers to cut, add, remove, or modify DNA sequences in various parts of the genome. It is defined as an adaptive immune system created by bacteria to protect themselves against foreign genomic materials such as phages and plasmids [21]. The CRISPR genomic sequence consists of spacer (transcribed into CRISPR RNA/crRNA) and repeat regions (transcribed into trans-activating crRNA/tracrRNA) and protein structure consisting of Cas components, and is categorized into different classes, types, and subtypes [22]. CRISPR/Cas system consists of a guide RNA sequence (gRNA) targeting a specific region of foreign genomic material and effector Cas (CRISPR-associated nuclease) proteins. gRNA sequences (containing tracrRNA and crRNA regions) can be designed according to the targeted region and provide a programmability feature to the CRISPR/Cas system [23]. Cas proteins are nuclease enzymes that recognize and cleave target sequences in DNA or RNA guided by the gRNA. The CRISPR/Cas system has become a technology that can be used for gene editing and molecular diagnosis, with the ability to target any gene region by programming the gRNA sequence.

In recent years, CRISPR/Cas technology has been used as a molecular diagnostic tool to detect both DNA and RNA with high sensitivity and specificity [24]. The most important effect of the CRISPR/Cas system for infectious disease detection was the identification of pathogens and nucleic acid-based diagnosis by using Cas proteins [25]. Diagnostic methods designed using the effector proteins Cas9, Cas12, Cas13, and Cas 14 have enabled the discovery of new approaches that are fast, highly sensitive and specific, cost-effective, easily adaptable, compact, and portable. While the Cas9 system has only target cleavage activity, Cas12, Cas13, and Cas14 systems additionally have non-target cleavage (collateral cleavage) activity upon target recognition [4]. By this activity, Cas12 and Cas13 cleave non-target single-stranded DNA (ssDNA) and single-stranded RNA (ssRNA), respectively. This allows for a variety of readouts (fluorescence or lateral flow) upon detection of nucleic acids via signal amplification and adding functionalized reporter nucleic acids that are also cleaved by collateral activity [26]. A wide variety of CRISPR/Cas diagnostic tools have been made by utilizing the target cleavage and/or collateral cleavage activities of Cas endonucleases.

The use of CRISPR/Cas technology in nucleic acid detection requires a preamplification or postamplification process. Therefore, it can be used for diagnostic purposes when combined with nucleic acid amplification methods. New diagnostic methods have been developed by integrating CRISPR/Cas technology with thermal cycler PCR as well as isothermal methods such as LAMP, recombinase polymerase amplification (RPA), nucleic acid sequence-based amplification (NASBA), and rolling circle amplification (RCA) [26]. According to the functions of the types of Cas endonuclease enzymes, including Cas9, Cas12, Cas13, and Cas14, PoC diagnostic tests were designed in which CRISPR/Cas technology was used in conjunction with various nucleic acid amplification techniques. Initially, a combined technology was created using the isothermal NASBA method in the preamplification process and the Cas9 enzyme that recognizes double-stranded DNA (dsDNA). CRISPR/Cas9 combined NASBA technology has been successfully applied for Zika virus detection and lineage discrimination as PoC diagnostic test [27]. CRISPR-associated reverse PCR (CARP) method, including the CRISPR/Cas9 process and postamplification process, was performed [28]. This technique was used to detect different human papillomavirus (HPV) subtypes. In a follow-up study, the CRISPR-Cas9 typing PCR (ctPCR) technique, which is a preamplification process, was applied for the detection of HPV subtypes [29]. Specific high-sensitivity enzymatic reporter unlocking (SHERLOCK) systems were designed by combining CRISPR/Cas technology using the Cas13 enzyme and RPA technique [30,31]. Diagnostic platforms were also created for viral pathogens, including dengue virus (DENV) and Zika virus (ZIKA), using the SHERLOCK method [31,32]. Cas14 enzyme was combined with the RCA method in the detection of microRNA (miRNA) and single nucleotide polymorphisms (SNP) [23]. CRISPR/Cas technology based on the Cas12 enzyme was used for pathogen-specific detection by integrating it with LAMP and RPA methods. This combined technology was initially named the DNA endonuclease-targeted CRISPR trans reporter (DETECTR) method [30]. Alternatively, CRISPR/Cas technology combined with PCR, termed a one-hour low-cost multipurpose highly efficient system (HOLMES), was also developed [33]. Many pathogen-specific studies have been conducted, and CRISPR/Cas combined LAMP technology has emerged following the development of the DETECTR method and its application to PoC diagnostic tests.

## 4. CRISPR/Cas Combined LAMP

**Figure 2:**
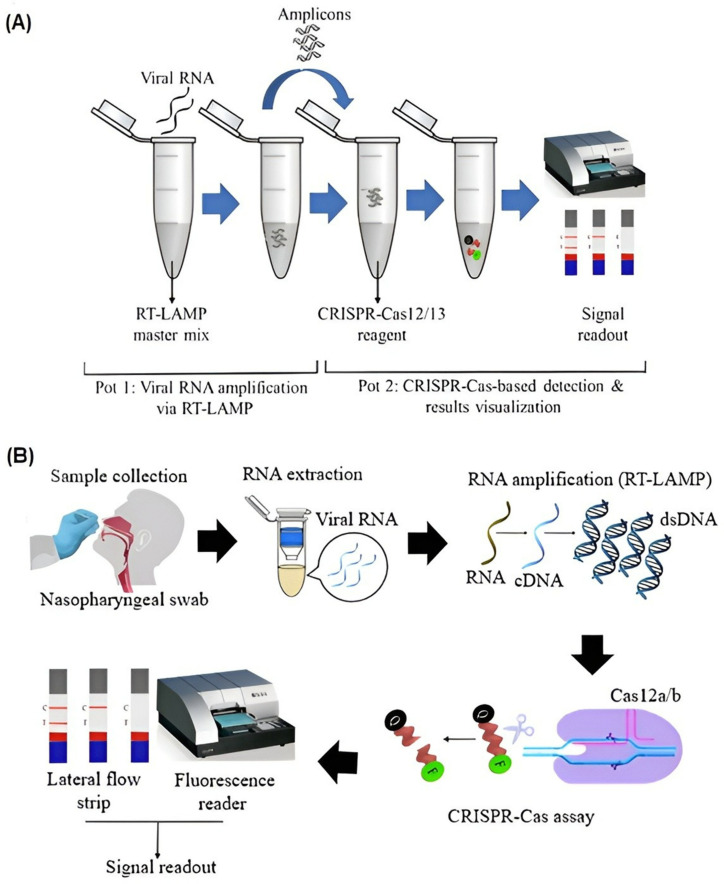
CRISPR/Cas technology. (**A**) Experimental process of CRISPR/Cas integrated LAMP technology [41]. (**B**) Schematic representation of the CRISPR/Cas combined LAMP diagnostic tests developed for the detection of SARS-CoV-2 [41]. Reprinted from Ref. [41].

CRISPR-integrated LAMP technology is a new generation nucleic acid amplification detection technology developed in recent years for pathogen detection [34]. It provides a tremendous opportunity to eliminate the limitations encountered during pathogen detection when both methods are utilized separately. LAMP technology is a method that has the capacity to rapidly replicate very small amounts of nucleic acid molecules in a short time. However, this advantage of LAMP brings with it certain limitations. As such, decreased sensitivity and false positive results are encountered due to several factors, such as contamination and primer-dimer formation [35,36]. Although the use of 6 primers in LAMP technology has aimed to achieve high specificity, the use of indirect detection methods focusing on the presence of the amplification product may produce false positive results [35]. In LAMP technology, detection is attained using turbidimetric, fluorescent, and colorimetric strategies, as well as agarose gel electrophoresis, which focuses on detecting the presence of double-stranded DNA after the reaction. Incorrect primer design, contaminated DNA, and non-optimal reaction components (pH indicators, interfering dyes) can lead to a decrease in specificity and sensitivity [37]. To overcome these problems, a sequence-specific detection method is required. CRISPR/Cas technology offers an advantage with its programmable nature and allows the use of reporters. It eliminates the limitations of LAMP and allows detection with high sensitivity and specificity [38]. When CRISPR/Cas technology is used directly for diagnostic purposes, an analytic limit of detection (LOD) value measured at the picomolar level range emerges [26]. The LOD value allows target detection when there is a high concentration of nucleic acid in the sample. The very high amplification capacity of the LAMP method, even at low concentrations of nucleic acid, easily provides the detection limit of CRISPR/Cas technology. In addition, after the CRISPR/Cas combined LAMP technology reaction is completed, the results can be monitored with fluorescent [39], visual [40], and lateral flow strips [34,41] (Figure 2A). This eliminates the need for quantitative measurement during amplification, as observed in other NAATs such as RT-PCR. The integration of these two technologies offers the opportunity to develop diagnostic tests with high efficiency, sensitivity, specificity, and no need for complex equipment. In CRISPR/Cas-combined LAMP technology, the Cas12 endonuclease enzyme was specifically preferred because of its collateral cleavage activity [26]. In the classification of Cas enzymes, Cas12 enzymes are known as type V and are categorized into class II [42]. According to the research carried out to date, many subtypes of Type V have been discovered, and a phylogenetic tree with 3 branches has been established [43]. The first branch includes Cas12a, Cas12c, Cas12d, Cas12e subtypes, while the second branch includes Cas12b, Cas12h, Cas12i. In the third branch, there are smaller effector subtypes, such as Cas12g. Among these subtypes, Cas12a and Cas12b have been used most frequently for CRISPR-combined LAMP applications.

Cas12 enzyme can gradually generate 5–7-nucleotide dsDNA breaks as it contains a single RuvC nuclease domain [44]. Cas12b enzyme requires a long sgRNA of 111 nucleotides containing crRNA and tracRNA regions [45]. The long sgRNA may cause partial overlap with LAMP primers in the targeted region and can lead to false positives. There are four or six LAMP primers, and primers are located close to each other on the targeted sequence according to the design principle. Cas12a enzyme requires a short gRNA of 41 nucleotides [45]. Therefore, the Cas12a enzyme is preferred in CRISPR/Cas combined LAMP technology. Originally, this was used to develop the DETECTR method, and various modifications were made for its pathogen-specific use and to improve the method. Different assay names, including HOLMESv2 [33], STOPCovid.v2 [46], and in vitro specific CRISPR-based assay for nucleic acids detection (iSCAN), were given [38]. The Cas13 enzyme has also been used in several studies to take advantage of its collateral cleavage activity in CRISPR/Cas combined LAMP technology [41]. However, since the Cas13 enzyme cleaves ssRNA, an additional T7 transcription step to convert DNA amplicons into RNA molecules after preamplification is required when Cas13 is utilized.

CRISPR/Cas combined LAMP technology has been used to detect various infectious viruses and bacteria (Table 1). Particularly following the outbreak of the SARS-CoV-2 pandemic, several CRISPR/Cas combined LAMP diagnostic tests have been developed to detect SARS-CoV-2 [41] (Figure 2B). Furthermore, amplification of the nucleocapsid protein, N, [46] or envelope, E, [38] gene regions of SARS-CoV-2 were achieved using RT-LAMP. Amplification products were then detected using Cas12a [38,39], Cas12b [46,47], or Cas 12 [34]. To ensure a simple, standardized, and user-friendly result analysis process, Cas-integrated LAMP-based assay analysis was achieved using lateral flow [34,48], or fluorescence [39,45]. For lateral flow-based tests, reporter molecules that could hybridize complementary test-line probes were used. In positive samples, upon Cas12 binding, the collateral cleavage activity would result in the cleavage of reporter molecules, altering their complementarity and, thus, producing different lateral flow readouts for positive and negative samples. For fluorescence-based tests, Cas12 proteins were used in conjunction with fluorescent quencher molecules, producing fluorescent readout for positive samples upon Cas12 collateral cleavage activity in positive samples.

A multitude of approaches was taken to develop sensitive, specific, user-friendly, rapid, low-cost tests. Furthermore, to reduce the duration of the extraction procedure and increase sensitivity, magnetic bead purification was integrated [46]. To minimize the cost of production and the assay turnaround time, a microfluidic chip was utilized, giving rise to a platform termed isotachophoresis (ITP) enhanced CRISPR (ITP-CRISPR) [49]. To detect emerging variants of SARS-CoV-2, a variant-specific amplification and detection platform was developed [50]. In addition to SARS-CoV-2 diagnostic tests, PoC platforms targeting other infectious viral pathogens, including Influenza A and B [53], Human Papillomavirus 16 and 18 [54], and Hepatitis C Virus [55] were also created using Cas12 or Cas13 integrated LAMP or RT-LAMP technologies. Similarly, pathogenic bacteria detection, including *Shigella flexneri* [40], *Neisseria meningitidis* [56], and *Klebsiella pneumonia* carbapenemase [57], was also achieved using Cas-integrated LAMP.

In many studies, it has been observed that this combined technology indicates up to 100% specificity and sensitivity in the range of 86–100% in pathogen detection [34,38,39,46]. Moreover, CRISPR/Cas combined LAMP technology was performed simultaneously with other techniques and compared in terms of sensitivity and specificity. For the detection of the African swine fever virus (ASF), the results of CRISPR/Cas12a and RT-PCR methods were compared in samples taken from different tissues [23]. CRISPR/Cas12a results were determined to be suitable as a diagnostic test in terms of sensitivity and specificity. In addition, it offered the possibility of detection in a shorter time compared to the gold standard RT-PCR. In another study, RT-PCR was performed simultaneously with the One-pot visual RT-LAMP-CRISPR system (opvCRISPR), designed for SARS-CoV-2 detection, and equal sensitivity and specificity were observed [25]. The assay turnaround time was very short compared to RT-PCR (120 min), producing results in 30–40 min, and is suitable to be used as a rapid test [25].

Although CRISPR/Cas combined LAMP technology was constructed to provide high sensitivity and specificity, it has a complex experimental procedure and some limitations [25,48]. As it was first designed, efforts have been made to reduce the complexity of this technology and overcome its limitations. The complexity stems from the fact that this technology requires two steps, the preamplification process and the CRISPR/Cas12 reaction process [26]. These two processes have different chemical components and reaction conditions, which brings about limitations [41]. The difference in the optimum temperature of the Bst enzyme used in the LAMP amplification and the Cas12 enzyme requires two different temperatures. In the HOLMESv2 assay, a one-pot reaction was achieved by using the thermophilic Cas12b (isolated from *Alicyclobacillus acidoterrestris* bacterium) enzyme (AacCas12b) [33]. The use of two different reaction processes and various chemicals can increase the risk of contamination as it requires multiple manual processes. Alternatively, to minimize the need for trained staff and the risk of contamination, single tube Cas integrated LAMP platforms were generated by sealing LAMP reagents with oil and adding CRISPR reagents on the lid [25]. A single-tube CRISPR/Cas12a enhanced LAMP (CRISPR/Cas12a-E-LAMP) was applied for the diagnosis of *Shigella flexneri* bacteria, which simplified the procedure for users and prevented aerosol contamination [40]. LAMP chemicals were placed at the bottom of the tube, and CRISPR/Cas12a chemicals were placed on the cap of the tube. After LAMP amplification, the tube was mixed upside down, and the CRISPR/Cas12a process was initiated. In the same method, visible detection was achieved by reflecting the fluorescent light of the ssDNA-FQ probe and the LED light.

CRISPR/Cas technology has also been combined with PCR and other isothermal methods, such as NASBA, RCA, and RPA, for diagnostic purposes [26]. PCR is not preferred in PoC diagnostic platforms due to the need for a complex device for thermal cycling. Similar to LAMP, other isothermal amplification methods also do not require complicated equipment. However, each has its own methodology and usually requires two primers, two or more enzymes, and special probes [58]. LAMP technology provides lower cost and convenience by using a single enzyme, differentiating it from these other methods. This feature reduces complexity and cost. It also provides higher specificity and higher efficiency with the use of multiple primers. For all these reasons, LAMP is preferred over other isothermal methods and is used more frequently. The challenge of advancing these next-generation CRISPR/Cas combined technologies to create more successful PoC diagnostic platforms remains. The methodology of each of the integrated isothermal amplification methods differs, and differences in strategies arise as a consequence of the distinct properties of Cas enzymes [26]. For this reason, CRISPR/Cas combined technologies are compared with the gold standard RT/PCR rather than with each other. Each isothermal amplification strategy has its own limitations that have not yet been overcome. For example, NASBA can only amplify RNA or single-stranded DNA using three enzymes. However, LAMP amplifies the double-stranded DNA molecule using a single enzyme. In addition, studies have shown that the sensitivity of the combination of CRISPR with NASBA is lower compared to other methods [26]. Usually, CRISPR/Cas technology is used in combination with RPA and LAMP [26]. RPA also converts the amplification product into an RNA molecule in the final step, and, therefore, it is often used in combination with the Cas13 enzyme. In LAMP, the amplification product is double-stranded DNA and is often combined with the Cas12 enzyme. The advantage of RPA is that it occurs at a low temperature close to the optimum temperature of Cas enzymes. The most important limitation is the low nucleic acid concentration in CRISPR combined RPA [59]. However, when CRISPR combined LAMP is used, a high concentration of amplification product can be obtained.

## 5. Point-of-Care Platforms

Due to their high specificity and high accuracy, NAATs are preferred over other techniques in the diagnosis of epidemic and infectious disease-causing pathogens. However, due to the exponential increase in the number of cases, especially during epidemics or pandemics, there is a need for faster, easier, less expensive diagnostic methods to eliminate the need for complex laboratory infrastructure. With the COVID-19 pandemic, the importance of PoC diagnostic platforms has emerged, and quite a variety of PoC diagnostic platforms are being developed following rapid technological advancements [38]. In this context, CRISPR/Cas combined LAMP-based diagnostic tests have been used to overcome some of the limitations of the NAATs as RT-PCR or LAMP [40]. Furthermore, CRISPR/Cas combined LAMP has become one of the promising new molecular approaches in pathogen detection, and studies in this field have gained great momentum in recent years. This combined technology was created to prevent false positives, which is a common shortcoming encountered when solely LAMP is used as a PoC diagnostic method [56]. Benefiting from the collateral cleavage activity of Cas enzymes in the CRISPR/Cas system, CRISPR/Cas integration into PoC platforms increases the sensitivity and specificity of the LAMP amplification product. With this activity, rapid detection can be achieved with fluorescent [52] and lateral flow strips [34], allowing the use of fluorescent and quenching reporters.

Microfluidic systems have emerged as powerful tools to operate small sample volumes, couple reactions on a single chip, or mimic biological phenomena. As such microfluidic technologies have given rise to micro-total analysis systems (μTAS) and lab-on-a-chip (LOC) models, which have been utilized for various applications ranging from paper-based microfluidics testing [60] to 3D tumor modeling [61] or POC pathogen diagnostics [62]. Specifically, the development of POC pathogen detection platforms has allowed for the miniaturization of amplification reactions, reducing the required sample volumes as well as the reaction reagents [63]. By making use of micro- and nano-fabrication techniques, several steps are coupled to take place on a single chip which significantly decreases the risk of contamination. Particularly, the use of isothermal amplification techniques in microfluidics-based POC devices has offered ease of design and less energy requirement compared to thermal cycling-based amplification strategies. In such platforms, the appropriate temperature is typically ensured using a small heating apparatus, and the amplification reaction takes place in a microchamber or microchannel. The reagents required for each reaction are stored in reservoirs on the chip, minimizing the need for experienced technicians to handle small volumes of expensive reagents. Separate channels can be used for detection purposes, and detection can occur using various methods, including fluorescence. While clinical sample preparation is often necessary before amplification can take place, various strategies have been employed to minimize sample handling, reduce the complexity of purification, and standardize the process. To achieve this, chemical, electrical, mechanical, and thermal cell lysis methods and microfluidic genomic material extraction strategies have been incorporated into μTAS [64]. Furthermore, microfluidic platforms have been modified to fabricate low-cost, easy-to-use, portable, highly sensitive, and specific POC diagnostic tools. For the detection of many pathogens using LAMP technology, a wide variety of PoC diagnosis platforms have been created using microfluidic discs [65], paper-based microfluidics [66], and devices that can be monitored using a smartphone [67]. Although CRISPR/Cas combined LAMP technology has been used in the last few years for pathogen detection, there remains a challenge to develop faster, low-cost, easy-to-use, and portable devices. Inspired by the PoC platforms used in LAMP technology, CRISPR-LAMP PoC devices typically rely on three stages of experimental processes: (1) nucleic acid extraction (DNA or RNA); (2) LAMP amplification process; (3) CRISPR/Cas reaction. Modifying these three steps, several PoC devices have been developed to detect various pathogens.

To perform all three steps in a single tube to maximize user convenience, various PoC diagnostic applications have achieved success. An in vitro Specific CRISPR-based Assay for Nucleic Acids Detection (iSCAN) was developed to detect SARS-CoV-2, in which chemicals were mixed so that the RT-LAMP reaction and the CRISPR/Cas reaction could occur in a single tube [38]. Alternatively, it was demonstrated that the LAMP amplification and CRISPR detection reactions could be carried out consecutively. The reaction was carried out at 67 °C in a single tube and produced successful readouts in 60 min. When two reactions followed each other, reactions were carried out at 67 °C and 37 °C, and results were detected in 45 min. In another study, SARS-CoV-2 detection was performed in a single tube by executing two reactions for 45 min [45]. However, RT-LAMP reaction chemicals and virus RNA were put at the bottom of the 0.2 mL tube. CRISPR/Cas12a chemicals were suspended on the cap of the tube. A heated block was kept at 67 °C for 30 min, with only the bottom of the tube in contact with the heat block. The cap of the tube was kept at 31 °C, and CRISPR/Cas12a chemicals were not affected by the temperature. After the RT-LAMP reaction, the tube was turned upside down; the chemicals were mixed and kept at room temperature for 10 min. Visual fluorescence detection was achieved by holding a UV lamp over the tube.

Research is also underway to incorporate the CRISPR/Cas combined LAMP technology into a microfluidic disc to fabricate novel PoC diagnostic platforms. CRISPR/Cas12 combined LAMP technology has also been integrated into an electric field-mediated microfluidic disc, termed Isotachophoresis (ITP) enhanced CRISPR (ITP-CRISPR) for SARS-CoV-2 testing [49]. To maximize the ease of use of PoC diagnostic tests, research is being carried out to ensure that the LAMP amplification stage and the CRISPR/Cas reaction stage, which occur at different temperatures, take place at a single temperature. In a study aimed at detecting SARS-CoV-2 variants, a Cas12b protein was expressed and isolated from the thermostable bacteria *Brevibacillus* sp. SYP-B805 (BrCas12b) was used [50]. Fluorometric detection was achieved by creating a BrCas12a one-pot detection assay at 62 °C. In addition, two different methods have been developed to ensure ease of use in fluorometric detection after the reactions are completed. In the first method, a battery-operated lens which reflects a UV-A blue light between 410–415 nm, and a mobile phone flashlight were used. The lens was integrated into the cell phone camera, and the cell phone flashlight was projected onto the PCR tube in a dark environment. Visual detection of the fluorescent radiation of FRET reporters was achieved. In the second method, the portable multiplexing detection prototype (FISSH) device was developed. This device was 22.86 cm × 26.66 cm in size and was designed to be easily portable, with a section for sample loading and fluorescent reading capability.

**Figure 3:**
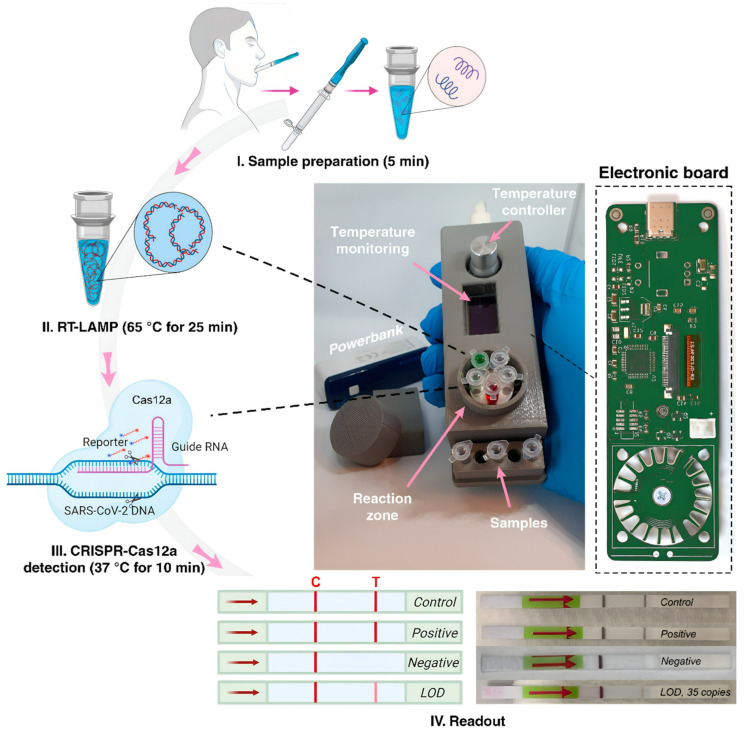
Schematic representation of the experimental process of CRISPR/Cas12a integrated RT-LAMP technique and portable PoC device [51]. Reprinted from Ref. [51].

To diagnose SARS-CoV-2, a portable device was developed as a PoC tool, in which the CRISPR/Cas12a combined LAMP method was utilized [51] (Figure 3). The rechargeable and semi-automatic device, with dimensions of 3.5 cm × 3 cm × 13 cm, was designed to be used easily in mobile laboratories, airports, and quarantine zones. It is capable of altering the temperature to ensure RT-LAMP amplification is conducted at 65 °C and CRISPR/Cas12a reaction at 37 °C. The device is suitable for ten samples and allows visual monitoring in the lateral flow strip. It is suitable to test 10 samples in 35 min in total and allows visual monitoring on a lateral flow strip.

## 6. Conclusions

CRISPR/Cas combined LAMP method is a next-generation technology developed for pathogen detection in the diagnosis of epidemic-causing diseases. Following advancements in technology in recent years, purpose-oriented strategies have been employed, and various methods have been integrated, giving rise to novel platforms that benefit from the existing methods. The COVID-19 pandemic and its damages have led to the development of such molecular combined technologies. It has also revealed that diagnostic tools are required to produce rapid, practical, high-efficiency, sensitive, and specific results. The use of complex laboratory infrastructure for diagnostic testing has further highlighted the need for user-friendly and field-usable PoC diagnostic methods and portable PoC devices. Therefore, there is a strong trend in the scientific community for the development of successful PoC diagnostic tests. To achieve this, there is an effort to improve existing diagnostic methods by using microfluidic systems, microchip technologies, microfabrication methods, and digital technologies.

CRISPR/Cas integrated LAMP technology is designed to create fast, highly specific, and highly sensitive PoC diagnostic tests. In this sense, it meets all expectations. However, it has not yet been fully adapted to the most successful PoC platforms. It is a complex and limited technology as it is a combination of two different molecular techniques. The struggle to alleviate this complexity and reduce the limitations still remains. Studies are carried out to ensure that all reaction processes of the method take place in a single tube [36] and to benefit from microchip technology [49]. On the other hand, studies are carried out to develop portable PoC devices that can be used at home, at the bedside, at airports, in border areas, and in quarantine zones [50,51]. As a result, CRISPR/Cas combined LAMP technology appears as a promising, highly efficient, and highly sensitive technology. Given the nature of LAMP technology and CRISPR/Cas technology, it is clear that this combined technology can be adapted to help develop successful PoC diagnostic platforms over time.

## Figures and Tables

**Table 1:** Advantages and limitations of pathogen-specific point-of-care platforms.

Method	Pathogen Targeted	Cas Protein	Advantages	Limitations	References
iSCAN	SARS-CoV-2	Cas 12a Cas 12b	High specificity (100%), single tube	Low sensitivity (50% for fluorescence, 86% for lateral flow)	[38]
SARS-CoV-2/DETECTR	SARS-CoV-2	Cas 12a	High sensitivity (100%) and specificity (100%), short assay time (40 min), single tube		[45]
STOPCovid.v2	SARS-CoV-2	Cas 12a	High specificity (98.5%)	Complex assay design	[46]
SARS-CoV-2 DETECTR	SARS-CoV-2	Cas12a	High specificity (100%) and sensitivity (100%), short assay time (40 min)		[48]
ITP-CRISPR	SARS-CoV-2	Cas 12a	Short assay time (30–40 min), high specificity (100%), reduced cost of production	Complex assay design, relatively low sensitivity (90.6%)	[49]
CRISPR-SPADE	SARS-CoV-2	Cas 12b	Short assay time (10–30 min), high specificity (99.4%), single tube assay design, variant-specific detection		[50]
Portable RT-LAMP/CRISPR	SARS-CoV-2	Cas 12a	Short assay time (35 min), portable device	Sensitivity and specificity no quantified	[51]
LAMP-LbCas12a Method	SARS-CoV-2	Cas 12a	Short assay time (40 min), high specificity (100%)		[52]
CRISPR/Cas12a Based Detection	Influenza A and B	Cas 12a	Relatively short assay time (75–85%)	Sensitivity and specificity no quantified	[53]
CIALFB	Human Papillomavirus 16 and 18	Cas 12a	High specificity (100%) and sensitivity (100%)		[54]
RT-LAMP Coupled CRISPR/Cas12	Hepatitis C Virus	Cas 12a	High sensitivity (96%) and specificity (100%)	Longer assay time (60–90 min)	[55]
CRISPR/Cas12a-E-LAMP	*Shigella flexneri*	Cas 12a	Short assay time (40 min), single tube	Sensitivity and specificity no quantified	[40]
CRISPR/Cas12a-LAMP	*Neisseria meningitidis*	Cas 12a	No false positives produced	Sensitivity and specificity no quantified	[56]
CRISPR/Cas12a-LAMP	*Klebsiella pneumonia* carbapenemase	Cas 12a	Short assay time (30–40 min)	Sensitivity and specificity no quantified	[57]

## Data Availability

Not applicable.
